# Territorial spillover of COVID-19 infections in Rome during the “second wave”

**DOI:** 10.3389/fsoc.2022.1066396

**Published:** 2022-11-14

**Authors:** Francesca Romana Lenzi, Francesco Giovanni Truglia

**Affiliations:** ^1^Laboratory of Psychology and Social Processes in Sport, Department of Movement, Human and Health Sciences, University of Rome “Foro Italico”, Rome, Italy; ^2^Italian National Statistics Institute (ISTAT), Rome, Italy

**Keywords:** spatial data models, COVID-19, urban health, β-convergence, socio-demographic effects

## Abstract

The study investigates the spread of the effects of COVID in 2019 in the city of Rome, focusing on the socio-economic factors that affect the incidence of the virus in the 155 urban areas (UAs) of the city. The units of analysis of this study are the UAs. The survey emphasizes the weight of spatial contiguity between the 155 UAs. For this purpose, the spatial data model analyses the spillover between contiguous units of analysis, distinguishing direct and indirect spatial effects. Digital geocoding of the collected data has been performed to create a geodatabase (GDB) that allows the statistical information to be turned into geographic layers. Geographic layers represent information layers that can be overlapped with each other on the map of Rome. The database allowed the variables to be handled with spatial analysis methods. This emphasizes the usefulness of digital analysis methods for the study of such a complex and rapidly changing phenomenon as the spread of SARS-CoV-19 infection on an urban scale.

## Introduction

A COVID-19 pandemic is an abnormal event, not fitting in the series of *usual* catastrophic events (natural disasters, conventional or non-conventional wars, etc.). First of all, it seems that, differently from the case of other catastrophic events, there is complete adherence between the meaning of the adjectives that describe this event and their empirical references. For instance, terms such as “global” or “world” have been used to label various tragic events including the two world wars, which, as a matter of fact, were not global in the sense of spatial extension.

The uniqueness of the present time, with the interruption of the monotonous, but reassuring, daily habits, seems to escape the focus of the studies on safety/unsafety in big cities. Suddenly, the frequently mentioned *liquid modernity* (Bauman, 2000) seems to have solidified and the social distancing, rather than weakening the importance of physical contact among people, proves that it is irreplaceable.

Obviously, since the pandemic is affecting our life in all its individual and collective dimensions, is relevant for different fields of study: from medicine to philosophy, epidemiology to sociology, and statistics to law. For instance, the interchange among these disciplines can be noticed in the overlapping of idioms and words that sometimes from being metaphors turn into real *discourses* on the government of life (Foucault, 2001). We owe the use and conceptualization of terms such as biopolitics and biopower to Foucault. In his view, a turning point in the evolution of the tools for the management of power is essentially marked by the techniques for the subjugation of human bodies through specific disciplinary devices (prisons, barracks, colleges, etc.).

In this way emerged the idea of the “microphysics of power” that collects and organizes the *laws* for the *treatment* of “naked life” or “sacred life,” to which G. Agamben devoted relevant studies (Agamben, 1995). A life that connects the biological dimension with the political one and that now, with the current crisis, is at the center of the political and philosophical debate^1^. Based on this perspective, it can be argued that the management of the pandemic can only be biopolitical. However, it has to be taken into account that under this term gather different formulations can be essentially divided into two groups. The first one is more markedly pessimistic or, as U. Eco described it, “apocalyptic” in which the now irreversible and pervasive aspects of the techniques of control and command are emphasized. The other one is more “optimistic,” where the life, the *bios*, seems to overflow the political dimension (Esposito, 2010). The reference to biopolitics is central, and the focus is placed in particular on the biopolitics approach with regard to two concepts: anomie and immunity.

By leveraging on these two concepts, an attempt has been made, if not to completely uncover, at least to open a crack from which to look at the socio-political and epidemiological interactions triggered by the COVID-19 pandemic. Interactions that bring to light terms, theories, and *knowledge* that have been systematized in the 70s of the XX century, but whose speculative layers date back to more ancient times.

At the same time, from an empirical point of view, the space–time dynamics of the spread of infections were analyzed, to seek to identify spatial continuity and discontinuity as well as time differences that characterize the “territorialization” process (Deleuze and Guattari, 1980) of the epidemic in the city of Rome. In addition, attention was paid to three segments of the population—a central element in Foucault's reflections on biopolitics^2^—which are the old-age index, the density of the population under 15 years of age, and the incidence of foreigners.

In conclusion, it has to be remarked that the *trait-d'union* between the two sections of this article is that common analytical dimension: the city.

## Materials and methods

### Space–time and demographic aspects of the spread of infections among the urban areas of Rome

Although the COVID-19 pandemic is much less lethal than other events of the same kind, it bursts into the XXI century and interrupts sociability—which no social substitute can replace—and imposes physical confinement and social distancing exactly as in past eras, thus eliminating relations between people, between people and places and even between places. The very short time within which the city, while remaining inhabited, emptied itself of social life, has been impressive. This experience certainly raises the awareness of the possibility that society itself can dissolve as well as of the potential speed of such dissolution.

Therefore, the duration and the speed of change appear to be the most distinctive characteristics of pandemics.

With regard to the first aspect, it is clear that with a prolonged duration of the “state of exception” the “shared values” and social cohesion weaken. There is no doubt that the effects of the pandemic, primarily the economic ones, affect different social strata in different ways, increasing inequalities^3^ and therefore the loss of trust in the institutions.

With regard to the second aspect, it is immediately evident that the speed of the spread of the virus corresponds to an equally sudden dismantling of social life. A dismantling that appears to be a sort of a strategic retreat aimed at cutting the supplies to the virus. Also in the case of the spread of COVID-19, as in all battles, the fighting concerns the defense of the territory.

In this study, rather than just the search for correlations between socio-demographic variables and infections—correlations that are nevertheless taken into consideration in the proposed models—an attempt has been made to focus the attention on the space–time dynamics of the spread of the virus and in particular, to take up the metaphor of the invasion, on the speed of its diffusion in the territory of Rome. In other words, the variable under investigation is not the effect of the change produced by the pandemic, but the speed of this change. Therefore, in this sense, the speed of diffusion can be understood as an indirect indicator of the timing of a social change in an urban context.

For this purpose, it is thus necessary to resort to models of spatial auto-regression, in which the growth of infections at time t_1_, in a certain Urban Area (UA), is related both to the level of infections at time t_0_ in the same UA and to the infections in the neighboring UA. The autoregressive aspect is hence informing us of the space–time dynamics, while the other socio-demographic and geographical indicators in the model have mainly the function of control variables.

Given the lack of individual data on the infections, it has been resorted to the analysis of aggregate data by UA. These territorial units by size, population, density, number of structural facilities, and so on, exceed by far the size of many Italian cities. Therefore, in this sense, Rome can be considered as a *city of cities* (Cipollini and Truglia, 2015).

From an operational point of view, the speed of diffusion can be reported by the rate of variation in the incidence of infections in the UAs. On the other hand, from a methodological point of view, it is possible to use β-convergence econometric models with spatial effects. As it will be explained more in detail below, it is a specific and diverse family of models for the treatment of space–time data that had been first used for the study of economic imbalances between different geographical areas (Taufer et al., 2016) and which, subsequently, had also been used in the socio-demographic and electoral fields (Truglia, 2019).

The data used in the research have been subject to a (digital) geocoding which allowed to generate variables that could deal with spatial analysis methods. This emphasizes the usefulness of digital analysis methods for the study of such a complex and rapidly changing phenomenon as the spread of SARS-CoV-19 infection on an urban scale.

The second section is divided into three parts. The first reports the basic data, their transformations, and a brief overview of the β-convergence model. The second is devoted to the description of the methodology of spatial regression models, highlighting their peculiarities. The third shows the results of the analysis.

### Basic information and re-coding

The data on the COVID-19 cases are collected by the Lazio Region—Epidemiology Department and refer to different periods of time between April 7, 2019 and November 2, 2020 (the latest data available at the time this study began).

For the purpose of the analysis undertaken below, in addition to the data on infections, it has been necessary to update to 2019 the information regarding the resident population, the population above 64 years and under 15 years old and the foreign population.

The data collected by the Municipality of Rome with the support of the Italian National Institute of Statistics (ISTAT) have been geocodified so that it has been possible to build a geodatabse that allowed to turn the statistical information into geographic layer. Each of these geographic layers amounts to an information layer that can be overlapped with the others on the map of Rome. This has allowed employing of statistical spatial analysis tools. The following indexes are calculated from the data on infections and those on the population:

— *incidence of infections* on October 5 and November 2 for each UA:
  $$\begin{array}{l} I_{\left(5,10\right)i}=\frac{N{}^{\circ}Infections\;on\;October\;5\;in\;the{\;}^{{\;}^{\prime }}i-th\;UA}{Population\;}\\ x10.000ab.; \end{array}$$
  $$\begin{array}{l} I_{\left(2,11\right)i}=\frac{N{}^{\circ}Infections\;on\;November\;2\;in\;the{\;}^{\prime }i-th\;UA}{Population\;}\\ x10.000\;ab. \end{array}$$
— *natural logarithm of the rate of variation in the incidence* of infections in the two periods of time considered (TVI) for each UA:
  $$\begin{array}{l} TVI_{i}=\ln\left(\frac{I_{\left(5,10\right)i}}{I_{\left(2,11\right)i}}\right)\\ =\ln\left(\frac{N{}^{\circ}infections\;on\;October\;5\;in\;the{\;}^{\prime }i-th\;UA}{N{}^{\circ}infections\;on\;November\;2\;in\;the{\;}^{\prime }i-th\;UA}\right). \end{array}$$
— *natural logarithm of the incidence of infections* as of October 5 (Ic), which for each UA is as follows:
  $$\begin{array}{l} Ic_{i}=ln\left[I_{\left(5,10\;\right)i}\right]. \end{array}$$

The other indexes, calculated for each UA, for which it is redundant to provide a statistical formulation and to which the role of explanatory variables is assigned are as follows:

— *The old-age index* (IV_i_). The effects of the COVID-19 infection can be particularly serious, with dramatic consequences, among the elderly who are the most fragile part of the population. For this reason, it is certainly important to investigate the relationship between the growth of infections and the levels of IV in the different UAs;
— *The percentage of foreigners in the population* (ST_i_). Although it was not possible to distinguish this variable by type of nationality, it seemed useful to include it in the analysis as it takes into account specific lifestyles connected to urban coexistence;
— *The population <15 years old per km*^2^ (D_i_). Among children from 0 to 15 years old, the number of asymptomatic people is significantly higher than in other demographic groups (Ismail et al., 2020). This peculiarity makes these individuals considerably effective carriers in the transmission of the virus in particular to the older generations; and
— *Infections as of November 2 per km*^2^ (C_i_). This is a context indicator entrusted with the task of highlighting the weight of the “community” on the countermeasures to contain the epidemic.

If not required by the statistical formulation, these indexes will be hereinafter referred to without subscript.

Furthermore, two geographical divisions have been recoded as *dummy* variables concerning, respectively, the localization of the UAs with respect to Gra (InGra/ExtraGra) and with respect to QSouth (QSouth/NoQSouth). The information on the geographical location of the UAs besides serving as control variables can also be a sort of a “container” of information on the socio-cultural and urban characteristics of the territory of Rome. A long tradition of studies has reported and formalized both from an empirical and theoretical point of view, the structural elements that characterize these areas of knowledge. For instance, consider the *diffuse city* and the *dense city* in which the extra-Gra and intra-Gra UAs respectively fall, or the UAs that form the part of Rome that extends in the QSouth between Eur and Ostia.

### Review and description of the data distribution

**Figure 1:**
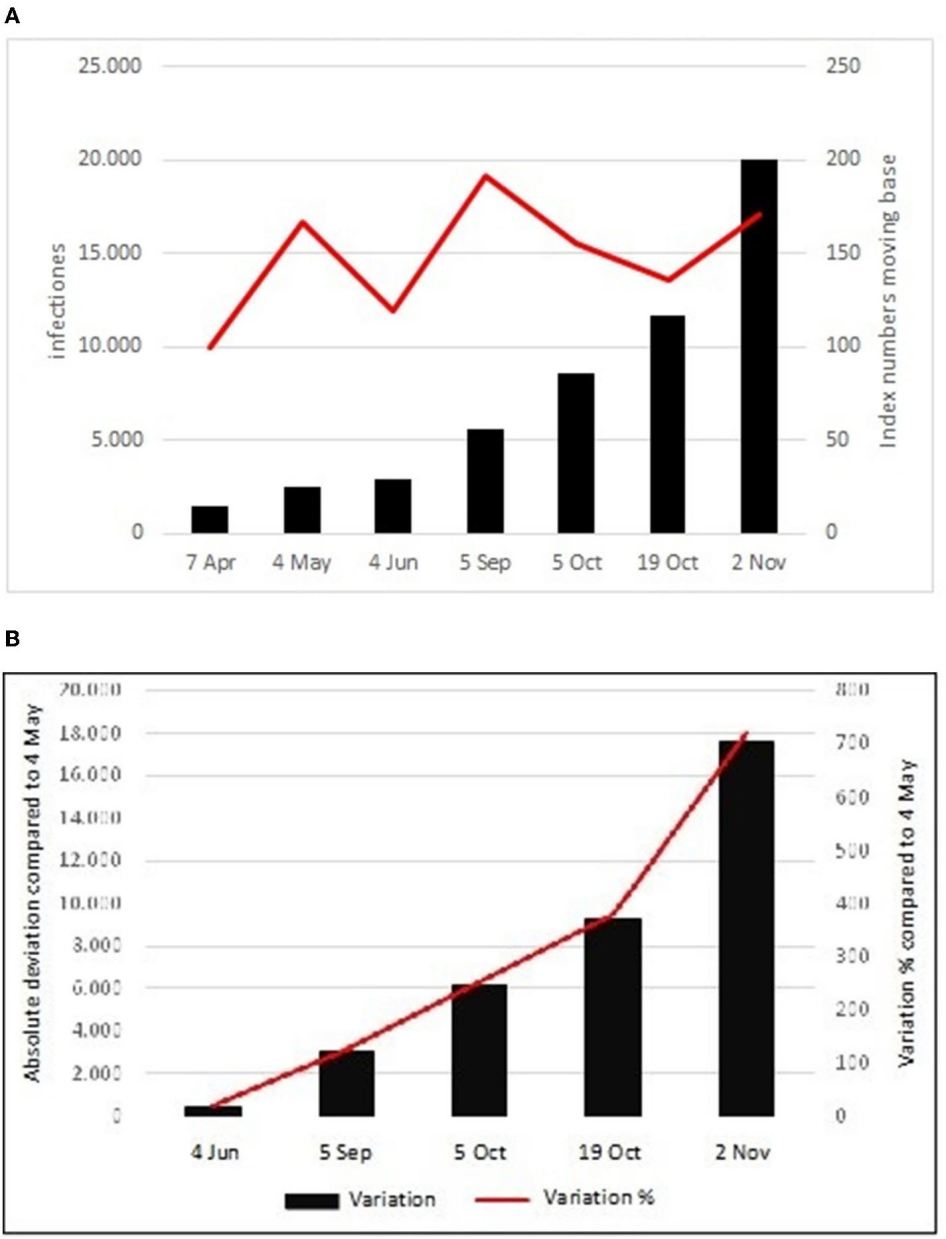
**(A)** Absolute values and index numbers with a moving base. **(B)** Absolute and percentage variation compared to the 4/5.

COVID-19 officially arrived in Italy on January 30, 2020, with the hospitalization of two Chinese tourists at the Spallanzani Hospital in Rome. Thus, it has been the capital, to begin with the interventions and health procedures for the treatment and containment of the pandemic in Italy. From January 30 to May 4, the cases of COVID-19 in the capital were 2,441. On June 4, 1 month after the end of the lockdown, the infections increased by 458 units and therefore stood at 2,899 (+185 compared to May 4). In the 3 summer months, the infected people increased by 2,639 units and stood at 5,538 (+126.9% compared to May 4). Starting from this date, we have witnessed a steady increase of cases which on October 5 reached the number of 8,612 (+252.8% compared to May 4) and on October 19 they exceeded 10 thousand, reaching a total amount of 11,711 infections (+379.8% compared to May 4). From this date, a strong acceleration has been registered, as highlighted by the change in the angle of inclination of the broken line (Figures 1A,B). On November 2, the total number of cases was 20,012 (+719.8% again compared to May 5).

**Figure 2:**
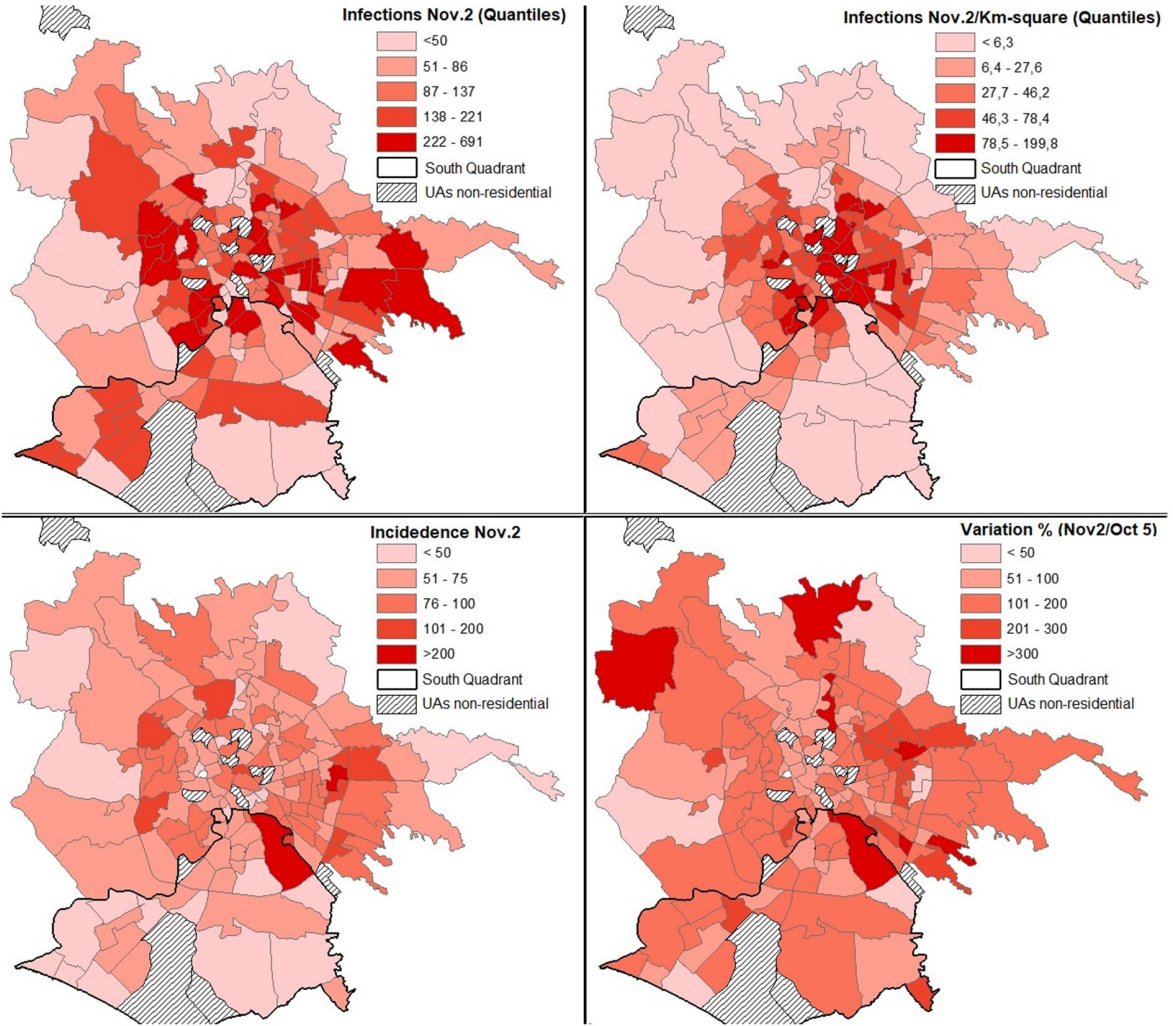
Territorial configuration of the infection indexes as of November 2, 2020.

From a territorial point of view, as of May 5, the UAs without infections were 20 (12 of which were non-residential), on October 5 they were reduced to 15 (of which 12 were non-residential), on November 2 the UAs in which no infections were reported were only two (both are non-residential). As of November 2, the 10 UAs with the highest number of infected were Torre Angela (691), Centocelle (497), Primavalle (436), Don Bosco (424), Tuscolano sud (369), Torpignattara (355), Gordiani (353), Trieste (343), Borghesiana (342), Gianicolense (323), and Esquilino (312) (Figure 2).

The geography of the pandemic shows a strong concentration of UAs with a high density of infections within the Gra (Figure 2). In fact, 20% of the UAs with the highest values are located in this territorial division (from 78 to 200 infections per km^2^).

With reference to the population, as of October 5, only one UA (Omo) has an incidence greater than 100 infections per 10 thousand inhabitants. A little less than a month later, on November 2, the UAs that exceed that threshold are 19, of which 12 are residential (Figure 2). Considering only the latter UA, from the data published on October 5, it appears that only Casetta Mistica has 0 infections and only the Omo UA has an Ic higher than 100. For the rest of the UAs, this number varies between 39.5 (Gregna) and 84.1 (Grotta rossa Ovest).

Considering only the residential UAs by incidence rate (a number of infections per 10 thousand residents), the most exposed, with increases of at least 150%, are Grotta Rossa Ovest, Casetta Mistica, Appia Antica Nord, and Omo. While the highest TVI (≥ 300%) are Santa Palomba, Navigatori, S. Maria di Galeria, Prima Porta, Barcaccia, Appia Antica Nord, Lucrezia Romana, Aeroporto dell'Urbe, and Tor Cervara (Figure 2).

**Figure 3:**
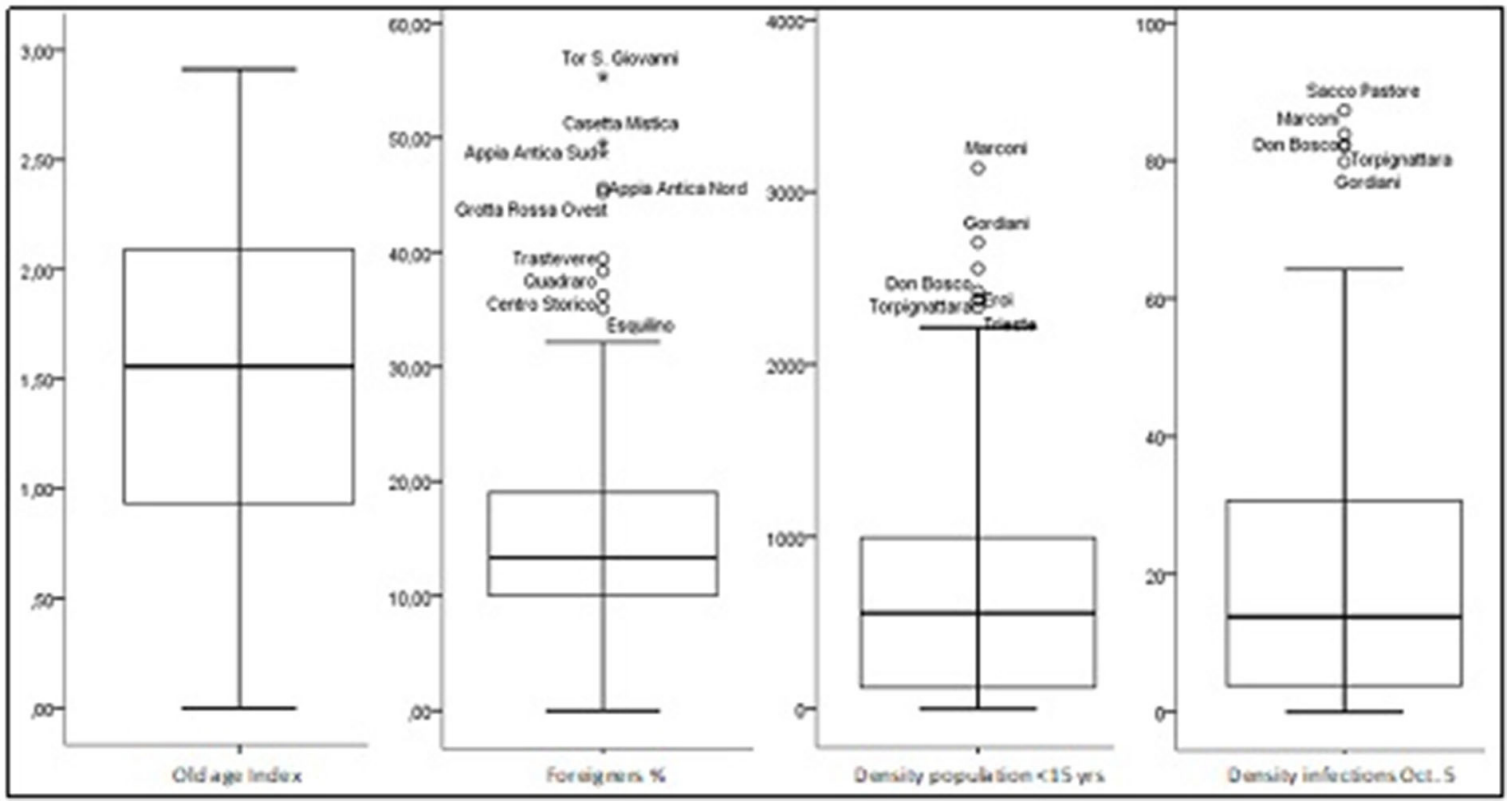
Characteristics of the territorial distribution of the explanatory variables.

With reference to the distribution of the other variables, the respective box plot (Figure 3) shows the following:

— A strong symmetry of the old-age index. In 50% of the UAs for every 10 children/young people affected there are at least 15 elderly people affected. The proportion is 10–30 in 25% of the UAs, the mean (M) and the coefficient of variation (Cv) are equal to 1.58 and 0.50, respectively;
— A marked right asymmetry in the density distribution of the foreign population. In 50% of the UAs, the percentage of foreigners is over 13.3%. There are nine UAs with a stronger presence of foreigners (at least 30%), and in three of them (Appia Antica Sud, Casetta Mistica, and Tor San Giovanni) the percentage is higher than 50% (M = 15.5; Cv = 0.65);
— A strong right asymmetry in the density distribution of the population under 15 years old. In 50% of the UAs, the value of this reference is greater than 556 inhabitants per km^2^. The UAs with the highest density of children/young people are six (Marconi, Gordiani, Don Bosco, Eroi, Tor Pignattara, Trieste). The mean and the CV are equal to 726,7 and 0,94, respectively; and
— A marked asymmetry in the density of infections reported as of October 5. In 50% of the UAs, there are at least 34 infections per km^2^. In four UAs, the value of this reference is greater than 75 infections per km^2^ (M = 46.3; Cv = 0.93).

**Figure 4:**
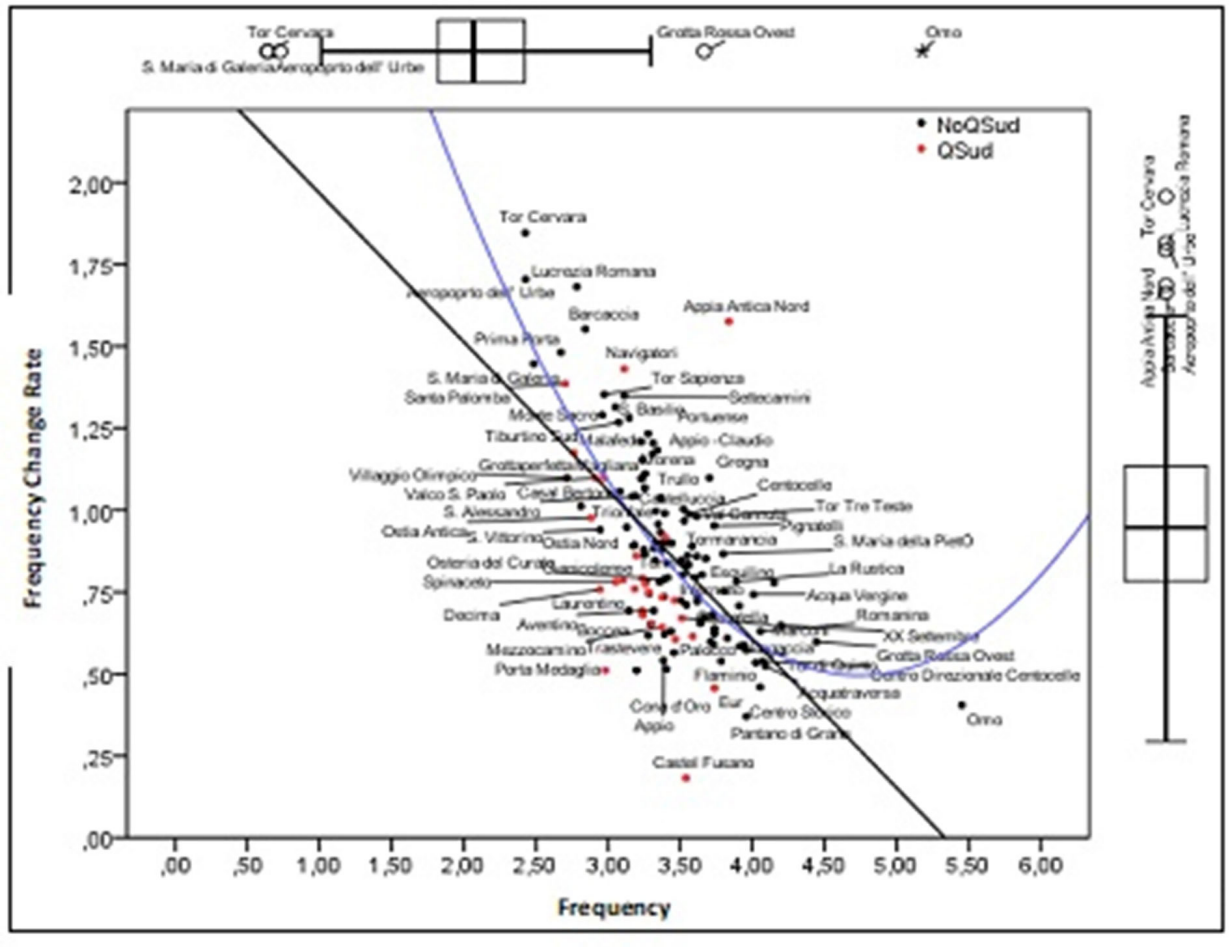
Univariate and bivariate features of the distribution of IC and TVI.

The distributions of incidence (Ic) and the rate of variation in incidence (TVI) are compared in the graphs presented in Figure 4 and which represent the starting point for the analysis developed in the following pages. The UAs with the lowest Ic values are Tor Cervara, S. Maria di Galeria and Aereporto dell'Urbe. The highest ones are Grottarossa Ovest and Omo. As regards the distribution of TVI, the UAs with the highest values, which are located above the “mustache” are Prima Porta, S. Maria di Galeria, Barcaccia, Aeroporto dell'Urbe, Appia Antica Nord, Lucrezia Romana, and Tor Cervara. Therefore, these are UAs that as of October 5 had low levels of Ic_i_.

The point cloud shows an inverse relationship between these two variables, whereby the TVI is higher in the UAs that had low Ic levels on October 5. This trend, therefore, signals the presence of territorial convergence.

### Urban balances and imbalances of COVID-19 infections: The β-convergence model

Among the different procedures for the analysis of territorial convergence (for which reference is made in the bibliography), in this study the β-convergence criterion was used in its simplest version and taking into account the transformations of the values into natural logarithms, has this function:


$$\begin{array}{l} TVI_{i}=\beta_{0}+\beta_{1}Ic_{i}+\;\varepsilon_{i}. \end{array}$$

The β-convergence model, therefore, reports whether in a certain period of time the territorial gaps, in relation to a specific aspect, increase or decrease; in this second case, there is territorial convergence with negative β_1_, while the speed of convergence is a function of this parameter (Arbia, 2005):


$$\begin{array}{l} \beta_{1}=-\left(1-e^{-bt}\right) \end{array}$$

from which it is derived as,


$$\begin{array}{l} b=\frac{\ln\left(1+\beta_{1}\right)}{t} \end{array}$$

where *t* is the length of the time period considered.

It should be emphasized that the term convergence does not mean a trend of the TVI toward the same level, but the achievement of a steady state that is specific for each UA (Alexiadis, 2010).

The trend of the point cloud seen in the scatterplot of Figure 4 shows a linear and a quadratic component that intercepts the variability due both to the UAs that are arranged in the upper section of the ordinate axis and to the single UA (Omo) which is located beyond the minimum point and for which there is no convergence.

In addition to the first-degree function, the parameters of a quadratic regression model were then estimated. In summary, the results report the parameter estimates (*p-value* < 0.01) of the quadratic linear regression model:


(1) $$\begin{array}{l} TVIi=2,35-0,33Ici+\varepsilon i\;R^{2}\;=\;0.39 \end{array}$$

(2) $$\begin{array}{l} TVIi=\;4,31-1,44Ici+0,15Ici^{2}+\;\varepsilon i\;Rc^{2}\;=\;0,47 \end{array}$$

The variance reproduced by the model [2] is almost 10 percentage points higher than that of the model [1]. This increase is probably due to the better representation capacity of the UAs that are arranged both along the upper section of the ordinate axis (UAs with the highest increases in the growth rate) and, at the same time, of the UAs with values of Ic higher than 4.42 (minimum point) which represents a threshold beyond which there is no longer convergence.

Over the period of time considered, which goes from October 5 to November 2, 2020, in just under a month, the number of infections went from 8,612 to 20,012 (+132.4%), the speed of convergence for the two models is equal to 0.12 and 5.3% daily, respectively.

### Econometric models for the analysis of spatial effects: Methodological aspects

Due to the “nature” of the analyzed phenomenon, a central role is played by the spatial links between the units of analysis. Therefore, it seems necessary to use specific procedures capable of considering the structure of the connections between the units of analysis (Guliyev, 2020).

In the cases investigated in this study, the mechanisms of the spatial spread of the COVID-19 pandemic are operationalized within an appropriate spatial regression model. A systematization of the models that adopt the econometric approach for the processing of spatial data is included in the works of Elhorst (2010) which are based on the methodologies developed by Anselin (1988) and Manski (1993). In a very simplified way, the spatial effects can be traced back to three types of interactions:

— Endogenous: the variation of the dependent variable (TVI) within an area depends on the change in the nearby areas;
— Exogenous: the variation of the dependent variable (TVI) within an area depends on the characteristics of the nearby areas; and
— Spatial autocorrelation: the change within an area of the dependent variable (TVI) depends on non-random factors, unobservable, or omitted variables in the nearby areas.

These three effects can be methodically organized in a general spatial regression model that can be formulated in the following way:


$$\begin{array}{l} y=\rho Wy+X\beta +WX\delta +u\;with\;\left|\rho \right|<1 \end{array}$$

(3) $$\begin{array}{l} \text{u}=\lambda \text{W}\text{u}+\varepsilon \;with\;|\lambda |<1. \end{array}$$

where:

— **y** is the vector (n^*^1) is the dependent variable (TVI);
— **X** is the matrix (n^*^k) of the explanatory variables;
— **u** is a vector (n^*^1) of regression residuals or errors due also to the presence of variables not explicit in the model;
— **W** indicates a matrix (n^*^n) in which the spatial structure of the units of analysis is organized based on a neighborhood criterion. For these analyzes, the criterion of the physical proximity of the units of analysis was adopted. So, two UAs are contiguous if they share at least one section of their boundary. The elements w_ij_ of the matrix **W** are equal to 1 or 0 depending on whether the *i*-th unit is contiguous or not to the *j*-th unit^4^;
— **Wy**, **WX**, and **Wu** are, therefore, three matrices (n^*^n) that represent the spatial *lag* of the dependent variable, the explanatory variables, and the term of error;
— **ρ** is the parameter associated with the spatial delay of the dependent variable and reports the endogenous effects of spatial interaction (*Spillover*). Therefore, it is an auto-regression parameter that measures the effects of the spatial delay on the same dependent variable;
— **β** is a vector (k^*^1) of regression parameters that report the influence of the explanatory variables on the dependent variable in the *i*-th UA;
— **δ** is a vector (k^*^1) that contains the parameters that measure the effects of the spatial delay of the explanatory variables (*Spinoff*). That is, the influence on the dependent variable in the *i*-th UA produced by varying the explanatory variables in the neighboring UA; and
— **λ** is the scale associated with the spatial delay of the regression error. It is, therefore, a measure of the autocorrelation of the error due to the omission of explanatory variables.

The formulation of the general model, therefore, depends on the rejection/acceptance of the null hypothesis on the value of each of these parameters. In a very simplified way, the spatial effects can be treated in autoregressive terms, thus using the *lag* of the dependent variable (Wy), or in terms of autocorrelation, through the error *lag* (Wu). In the first case, there is a Spatial Autoregressive Model (SAR), in the second a spatial error model (SEM). Finally, it is possible to treat both the delay error and the dependent variable (SARMA) at the same time.

The general model can be formulated in a single expression in the following manner (Florax and Folmer, 1992):


(4) $$\begin{array}{l} y=\rho WY+X\beta -WX\delta +{\left(I-\lambda W\right)}^{-1}u \end{array}$$

which with a few simple steps can be written in the following way:


(5) $$\begin{array}{l} y={\left(I-\rho W\right)}^{-1}\left(I\beta +W\delta \right)X+{\left(I-\lambda W\right)}^{-1}u. \end{array}$$

This formulation makes it possible to clarify the mathematical, and therefore interpretative, peculiarity of the regression parameters associated with the explanatory variables and their spatial delay.

Unlike what happens in OLS models, the relationship of dependence between the dependent variable and each independent variable (marginal effects) is not indicated by the usual coefficients β_r_, but from the s_ij_ elements that make up the matrix **S** (Le Sage and Pace, 2009):


$$\begin{array}{l} S=\left[\begin{array}{ccc} s_{11} & \cdots & s_{1n}\\ \vdots & \ddots & \vdots \\ s_{1n} & \cdots & s_{nn} \end{array}\right]\;with\;s_{ij}={\left(I-\rho W\right)}^{-1}\left(I\beta_{k}+W\delta_{k}\right). \end{array}$$

The information contained in the matrix **S** takes into account the peculiarity of the analysis of the spatial series compared to the temporal ones. For the latter, in fact, the dependent variable at time *t* is related only to the same variable reported at time *t-1* and the interaction is unidirectional (time *t* cannot affect time *t-1*). In spatial models, the relationship is multidirectional, in the sense that the variation of *y*_*i*_ influences and is influenced by the variations that occur in the nearby units.

In other words, the infections in a given UA(a) can produce variations of the infections in the neighboring UAs which, in turn, can affect the variation of infections in the same UA(a).

The spatial effects, organized in the matrix **S**, are therefore of three types:

— Direct (EI): impact of the explanatory variables on the *y*_*i*_ in the same unit area. These effects are indicated by the coefficients *s*_*ij*_ that are arranged on the diagonal of the matrix (i=j), thus from the trace of the matrix tr(**S**). So by dividing tr(**S**) by the number of units (n) we obtain the direct average effects (EMD);
— Indirect (ED): impact on the variable *y*_*i*_ in the *i*-th due to the variation of the explanatory variables in the zones close to it. These effects are given by the sum of the elements *s*_*ij*_ outside the main diagonal of the matrix (i ≠ j). By dividing this sum by the number of zones we obtain the indirect average effects (EMI); and
— Total (ET): impact of all *s*_*ij*_ elements (ET = EI+ED). Also in this case, the total average impact (IMT) is obtained from the division by the number of units, which can be interpreted in two different ways: i) effects produced by the *i*-th zone *on* all the others (sum of the elements of the *i*-th row of matrix **S**); ii) effects produced on the *i*-th zone *by* all the other zones (sum of the elements of the *j*-th column of the matrix **S**).

The process of identifying the most suitable model to study spatial effects is divided into several steps which, in a very schematic way, can be summarized as follows:

1. OLS model estimate;
2. test (Anselin, 1988) on the spatial independence of the residues and based on the results choice of the SEM or SAR model;
3. assessment of the parameters with the maximum-likelihood (ML) method;
4. test for the inclusion of exogenous variables in the model both in direct and delayed form; and
5. analysis of the suitability of the model to the data.

By setting ρ = λ = δ = 0, therefore eliminating the variables, from the model [3] we obtain the following multiple regression:


(6) $$\begin{array}{l} \text{y}=\rho \text{W}\text{Y}+\text{X}\beta \end{array}$$

where the matrix **X** contains the explanatory variables.

The estimates of the OLS parameters represent the first step for the analysis of the spatial effects and are shown in Table 1 together with those of the SDM.

**Table 1:** Figures and tests for the identification of the model.

**Test**	**Figure**	**Df**	***p*–value**
Moran	0.158		0.0025
LM-error	8.780	1	0.0030
LM-lag	10.02	1	0.0015
RLM-error	1.028	1	0.3107
RLM-lag	2.269	1	0.1319
SARMA	11.051	2	0.0040

Starting from the OLS model, the analysis is carried out to verify the presence or absence of spatial dependence of the residuals and to identify the most appropriate procedure for their statistical analysis.

The Moran index, which registers the presence of spatial autocorrelation on the residues produced by the OLS model, is approximately 0.15 and is statistically significant (*p*-value = 0.0025). The outcomes of the diagnostic tests point in favor of an autoregressive model with the spatial delay of the dependent variable (SAR). In fact, although the LM-error and LM-lag tests are both significant, the latter is associated with a higher test statistic and therefore a lower *p*-value.

Furthermore, the tests carried out on the external variables recommend this information to be entered both in the direct form and as a spatial delay.

The choice of an autoregressive model, which also incorporates this information, is also supported by the rejection of the null hypothesis of the *Common Factor Test* (*likelihood ratio* = −403.18; df = 7, *p*-value = 0.000)^5^.

The econometric model that best lends itself to the treatment of the spatial dimension of TVI, therefore, is the *Spatial Durbin Model* (SDM) which can be formulated as follows:


$$\begin{array}{l} \text{y}=\rho \text{Wy}+\beta \text{X}+\delta \text{WX}+\text{u}\;with\;|\rho |<1. \end{array}$$

## Results

### Effects of the socio-spatial interactions on the spread of COVID-19

The estimate (ML) of the parameters of the SDM model together with those of the OLS estimates are also reported in Table 2, which is divided into two sections. In the first section, in dark gray, there are the estimates of the regression parameters β_k_ which report the non-spatial effects of the explanatory variables on the dependent variable. In the second section, in light gray, the parameters that account for the spatial effects (Spillover and Spinoff) are organized.

**Table 2:** Estimates of the parameters of the OLS and SDM model.

		**OLS**				**SDM**			
		**Estimate**	**Er. St**.	**t**	**Pr(>|t|)**	**Estimate**	**Er. St**.	**t**	**Pr(>|t|)**
βk	Constant	4.281	0.408	10.505	0.0000	2.813	0.927	3.036	0.0024
	Incidence (Ic_i_)	−1.285	0.228	−5.640	0.0000	−1.389	0.193	−7.185	0.0000
	Incidence^2^ (${\text{Ic}}_{\text{i}}^{2}$)	0.119	0.032	3.757	0.0003	0.130	0.027	4.874	0.0000
	Old–age index (IV_i_)	−0.087	0.027	−3.225	0.0016	−0.068	0.027	−2.488	0.0129
	Foreigners %(St_i_)	0.000	0.001	0.269	0.7884	0.002	0.001	1.531	0.1258
	Infections on November 2 per km^2^ (C_i_)	0.005	0.001	4.994	0.0000	0.004	0.001	4.940	0.0000
	Density < 15 years (D_i_)	−0.000	0.000	−4.885	0.0000	−0.000	0.000	−4.775	0.0000
	Qsouth vs. No Qsouth (QSouth_i_)	−0.076	0.029	−2.580	0.0110	0.164	0.058	2.842	0.0045
	InGra vs. ExtraGra (Gra_i_)	0.114	0.034	3.332	0.0011	0.014	0.041	0.341	0.7330
ρ	Lag-growth rate (WTVI_i_)					0.287	0.111	2.576	0.0010
δ_k_	Lag-Incidence (WIc_i_)					0.652	0.420	1.551	0.1209
	Lag-Incidence^2^ (${\text{WIc}}_{\text{i}}^{2}$)					−0.064	0.057	−1.118	0.2636
	Lag-old-age index (WIV_i_)					−0.091	0.054	−1.695	0.0901
	Lag-Foreigners %(WSt_i_)					−0.007	0.003	−2.714	0.0067
	Lag-Infections on November 2 per km^2^ (WC_i_)					0.000	0.002	−0.113	0.9102
	Lag-Density < 15 years (WD_i_)					0.000	0.000	0.216	0.8290
	Lag-QSouth (WQSouth_i_)					−0.245	0.065	−3.774	0.0002
	Lag-InGra (WGra_i_)					0.196	0.068	2.857	0.0043

The main differences between the two models concern [6] and [7], the significance of the parameter associated with the geographical repetition InGra/ExtraGra, and the percentage of foreigners who are no longer significant in the SDM. For the other parameters the following considerations can be made as follows:

— Compared to the OLS model, the parameter associated with the IC remains negative and with lower values, indicating, as already mentioned above, the presence of territorial convergence reported by all models. This is stronger in the SDM in which the daily convergence rate is equal to 3.2%.
— The parameter associated with the quadratic term that reports the non-linear effects between Ic and TVI remains positive. For values of ${\text{Ic}}_{\text{i}}^{2}$ lower than 7.72, this increase is very limited, while it increases noticeably for values above this threshold;
— Even in the SDM, the relationship between the old-age index and the TVI remains negative. The latter is on average lower in the UAs with a higher prevalence of elderly people;
— The percentage of the foreign population is still not significant, even though the *p*-value has dropped considerably compared to the OLS estimates. Although the parameter associated with this variable is not significant, it seems interesting to underline the positive relationship between the increase in the percentage of the foreign population and the rate of variation;
— The density of infections as of November 2 and the density of the population under the age of 15 years, albeit with very low values of the regression coefficient, have a statistically significant influence on the TVI which increases as the first indicator increases and decreases in the UAs with a high density of children and young people; and
— In the UAs of the QSouth the levels of the TVI are lower than in the rest of the city. While the division InGra/ExtraGra has significant effects only in the OLS model (in the UAs within the Gra the TVI are higher).

The significant spatial effects are as follows:

— *Spillover*: The variable of autoregression ρ, just below 0.30 in fact indicates the presence of spatial dependence between the units of analysis. In other words, increases/decreases in the TVI in the *i*-th UA are also attributable to the variations of the same variable in the neighboring UAs.
— *Spinoff* relating to: *i)* the old-age index (WIV_i_). The UAs with a high presence of elderly people negatively affect the TVI of the neighboring UAs (containment effect); *ii)* the percentage of foreigners (WSt_i_). UAs with low TVI are close to UAs with high percentages of foreigners. Therefore, it would seem that the UAs with a high rate of foreigners curb the growth of infections in the neighboring areas; *iii)* the two geographical partitions (lag-InGra and lag-QSouth)^6^. The TVI decreases with the increase in the proportion of the UAs of the QSouth neighboring the *i*-th UA and, conversely, increases with the increase of the intra-Gra UAs neighboring the *i*-th UA.

Finally, it seems interesting to note that the spatial delay of the incidence (WIc_1i_), although not significant (the *p-value* is just over 10%), can, however, give some indications on the spatial mechanisms of the transmission of infections. It would seem, in fact, that the increase in the incidence in the UAs produces an increase in the rate of variation in the neighboring UAs.

Before concluding this section, it is necessary to come to some methodological considerations, in substantive terms, on the interpretation of the regression coefficients β_k_ associated with the explanatory variables which, as already explained above, can have direct, indirect, and total impacts on the dependent variable (Table 3).

**Table 3:** Breakdown of the spatial effects.

	**Impact**		
**Index**	**Direct**	**Indirect**	**Total**
Incidence (Ic_i_)	−1.3728***	0.3395	−1.0333°
Incidence ^2^ (${\text{Ic}}_{\text{i}}^{2}$)	0.1286***	−0.0353	0.0934
Old–age index (IV_i_)	−0.0748**	−0.1482*	−0.2230**
Foreigners % (St_i_)	0.0016	−0.0092**	−0.0076*
Infections on November 2 per km^2^ (C_i_)	0.0041***	0.0013	0.0054*
Density < 15 yrs (D_i_)	−0.0003***	−0.0001	−0.0003°
Sign.	0.001***;0.01**;0.05*;0.10°		

The number of * indicates the levels of statistical significance.

The statistically significant impacts of the Ic are direct and total ones and both negative, which implies that if the IC increases by 1% in the *i*-th UA, the TVI decreases by 0.14% in the same UA. The same considerations apply to the total impact that highlights the presence of negative global interaction: an increase of 1% of the Ic in all the other UAs produces a decrease of 1.03% of the TVI in the *i*-th UA.

Therefore, by following this outline it is possible to interpret the marginal effects of the different variables both with respect to the type of impact and with respect to the relationship (positive or negative) that each of them has on the rate of variation of the infections. In particular:

— In quadratic terms only the direct impact is significant and is in a positive relationship with the TVI;
— The impacts of the IV are all significant and positive. Therefore, an increase in this index in the *i*-th UA, or in the neighboring UA, produces a decrease in the TVI in the *i*-th UA;
— The foreigners have a negative, but not significant, direct impact, while the indirect impacts are negative and significant: the TVI in the *i*-th UA decreases with the increase in the percentage of foreigners in the neighboring UAs and, in general, in all UAs of the city;
— The impacts of the number of infections per km^2^ (C) are all positive, but only the direct and total ones are significant. As the density increases in the UAs neighboring to the *i*-th UA and in all the UAs, there is an increase in the TVI in the *i*-th UA; and
— The impacts of the density of the population under the age of 15 years (D) on TVI are all negative and significant.

It is worth remembering that the basic hypothesis of spatial regression models is that the units of analysis are not independent of each other, so it is not correct to estimate their parameters with the OLS method, as in traditional regression models. This method, in the case of spatial dependence, produces inconsistent estimates. For this reason, it is necessary to resort to other methods, including that of ML. Therefore, it follows that the R^2^ index is not suitable for assessing the suitability of spatial regression models whose parameters are calculated with different criteria than the OLS.

Therefore, in this case, the suitability of the model is evaluated by comparing the figures of AIC, of the log-likelihood (LogLik) calculated for the different models (Table 4).

**Table 4:** Figures on the suitability.

	**df**	**AIC**	**LogLik**	**L–Ratio**	***p*–value**
OLS	10	−165.91	92.955		
SDM	19	−193.86	115.931	45.952	0.0000
SEM	12	196.77	−86.384		
SDM	19	−193.86	115.931	404.63	0.0000
SAR	11	−172.74	97.372		
SDM	19	−193.86	115.931	37.119	0.0000

The AIC for the MDS is getting lower and lower. Conversely, the LogLik values are higher. The differences between these figures are statistically significant; therefore, the SDM model is the one that best fits the data.

### Territorial and socio-demographic aspects of the spread of COVID-19

As already mentioned, the socio-demographic indexes in the econometric model play the role of variables and control. However, the dramatic impact of the pandemic crisis on the elderly and the steady incidence of asymptomatic cases among children and young people suggested that a study should be dedicated to these two aspects. For this purpose, the first element of evaluation concerns the structure of the territorial configuration of the distribution of the three indexes (TVI, IV, and D) which was investigated with the Moran index (Moran, 1950). Having ascertained the presence of spatial autocorrelation, the (high/low) levels of TVI were associated with the (high/low) levels of the old-age index and with those of the density of the population under 15 years old, calculated based on the respective average values.

The intensity of the autocorrelation^7^ is higher for the IV (0.380) and followed by the index D (0.371) and TVI (0.211). These values are statistically significant (*p-value* = 0.01) and indicate the presence of a territorial aggregation process. In other words, contiguous UAs tend to have the same values on each of the three indexes.

**Figure 5:**
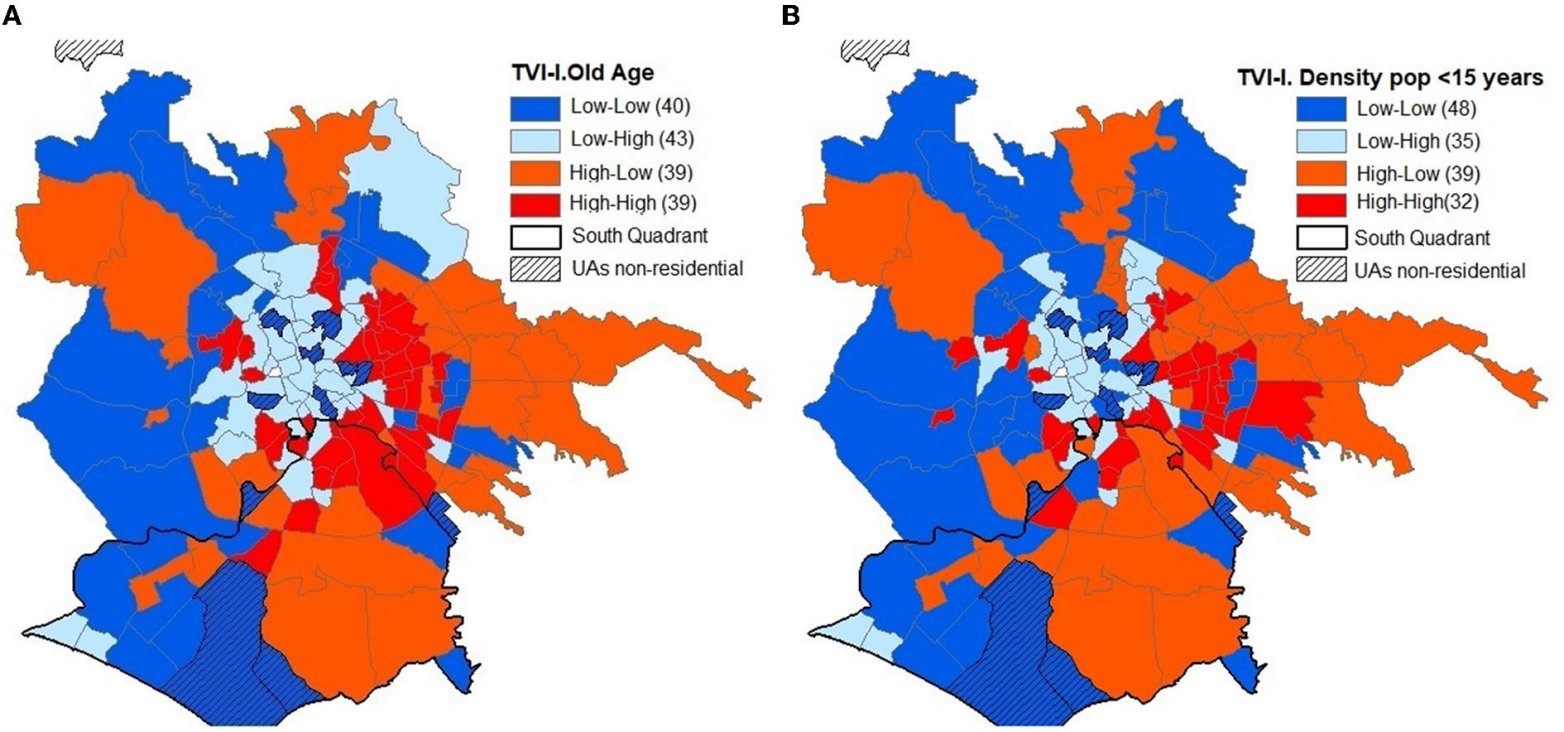
**(A)** Spatial and territorial structure between TVI and IV. **(B)** Spatial and territorial structure between TVI and D.

The second step concerns the territorial configuration of the combinations between TVI and IV (Figure 5A) and between TVI and D (Figure 5B) whose colors reflect the following combinations:

- blue → UAs with low TVI and low IV or D values;
- light blue → UAs with low TVI and high IV or D;
- orange → UAs with high TVI and low IV or D; and
- red → UAs with TVI and IV or D high.

The territorial configurations of TVI-IV and TVI-D are very similar. Excluding Martignano, of the 154 UAs, 119 have the same properties on the two combinations (diagonal of Table 5).

**Table 5:** Distribution of the UAs according to the combinations between the levels of TVI-D and TVI-IV.

	**TVI-IV old age**				
**TVI-Density pop < 15yrs**	**Low-Low**	**Low-High**	**High-Low**	**High-High**	**Total (TVI-D)**
Low-Low	36	12			48
Low-High	4	31			35
High-Low			26	13	39
High-High			6	26	32
Total (TVI-IV)	40	43	32	39	154

There are 39 UAs with high levels of TVI-IV and, with the exception of Spinaceto, they are all located within the GRA mainly between the center and the periphery of the ring QEast and QSouth.

The UAs with high level of TVI and low level of IV are 32 and comprise mainly the UAs of the extra-ring periphery of the QEast and the QSouth.

The areas with high levels of TVI and IV are 39 and, with the exception of Spinaceto, all are located within the GRA. Of these UAs, eight are located in the QSouth (Appia Antica Nord, Grottaperfetta, Laurentino, Navigatori, Spinaceto, Tor Marancia, Tre fontane, and Valco San Paolo) and 20 in the QEast.

The UAs with low levels of these two indexes are 40 and are arranged from South to North between Castelporziano and Cesano.

The UAs with low-TVI and high-IV levels are 40 and form a large cluster including the central western intra-Gra UAs.

The territorial configuration based on the combination of TVI-IV levels largely mirrors that of TVI-D, with the exception of 35 UAs and those that regard, in a more consistent way, the low–low levels, those that increase by eight units, those that regard the UAs of the intra-Gra and QWest periphery, those extra-GRA of the QNorth and high–high which decrease seven units that are located inside the GRA in the QEast and QSouth.

## Discussion

While completing the chapters on home safety and road accidents, where we have tried to describe and highlight some aspects of lack of safety in UAs during the second modernity, other elements of lack of safety, those descending from the COVID-19 pandemic, broke into everyday life, upsetting it and bringing back fears and scenarios that seemed not possible to come to existence.

Among the contradictions that the socio-health crisis has highlighted, at least for the Western world, is certainly the one between public alarm and home safety. If on the one hand, on the public side, and in particular on the health side, the mass media show dramatic and, in many cases, tragic scenes, on the other hand, on the private side, time seems to be suspended in the *unsafe* comfort of the daily life.

In the case of the COVID-19 pandemic, the lack of safety is not triggered by the usual “threats” associated with the second modernity but appears to be due to the very irruption of nature itself into the second modernity. A small virus was enough for the possibility of “naked life” to take a substance and reveal the fragility of society and the power of nature. Givone writes in *Metaphysics of the plague*: “One would think that the civilization, with its frames of values and disvalues, with its sophistication, its delights, and even its perversions, is nothing more than a false construction destined to collapse at the first encounter with the reality” (Givone, 2012; p.16).

The images of the empty streets and squares during the lockdown show the *bare city*, in which the grandeur of monuments and architecture reinforces the absence of man. Those images refer to the metaphysical squares of De Chirico's paintings, which in years of another virus, that of the warlike and racist ideas of fascism, infected the population and at the same time immunized it from the basic principles of freedom and equality.

In *Immunitas*, Esposito proposes a possible solution to the community-immunity antinomy, and seems to find it right within the functioning of the immune system and in particular in the “immunological tolerance.” He suggests an overlap between the language of medicine and that of politics, which thus shows deeper and more lasting bonds and biopolitical contiguities than previously thought. Esposito writes: “but perhaps it is precisely the figure of the implant – artificial as a prosthesis or natural as a fertilized egg in the mother's womb – to provide the most powerful evidence. The fact that it is precisely the genetic heterogeneity – and not the similarity – of the fetus that favors its acceptance by the woman's immune system means that this cannot be reduced to a simple function of rejection toward the stranger. But if anything, it should be interpreted as its internal sounding board, as the diaphragm through which the difference involves us, and passes through us, as such” (ivi, p. 18).

If this is the case, then immunization is both protection and denial of life and the fact that this second aspect has been given prominence, so to speak, is the consequence of a precise political choice that has put fear at the center of its discourse. First of all, fear of the different, fear of the new. Fear that the space of social relations is the premise of the return to the “state of nature” and therefore it should be harnessed and immunized. It is, therefore, a question of emphasizing the positive aspects of immunization starting with the development of a new semantics. For example, think of the term “body” used to indicate the *embodiment* of the state, of society, of a political apparatus. This translation seems to have lost its metaphorical character to acquire a more substantial and institutional one^8^. The consequence is that if the state is a body, if the electorate is a body, if the society is a body, and so on, then it is possible to *cure* them using those specific methods and therapies to heal the body. In other words, the use of this language brings with it the immune logic of biopower as analyzed by Foucault.

The current socio-health crisis, with its political–legislative implications, allows us to *test* the effects of the pandemic on the social arrangements and gives us the opportunity to rethink and update our views of government mechanisms. As is the case with the virus, biopolitics is also subject to change and can be rethought by removing and *immunizing* it from the *thanato-political* drift to bring it closer to the lands of *biopolitics*. In this changed horizon, then, biopolitics stands as a protection of the “law of the gift”^9^ which is the *nomos* of common life.

The countermeasures to contain the effects of the epidemic have eliminated social relations and transformed deeply rooted habits. However, as already mentioned, the most impressive aspect is the speed with which this change took place and which reveals how delicate, in their simplicity, the mechanisms of living in common are. At the same time, the duration of the pandemic underlines the irreversibility of this change. It is on these two aspects, speed and duration, that the empirical analysis of XXI century metropolis has been based.

From a statistical–methodological point of view, the possibility of using specific econometric models to investigate some space–time mechanisms that govern the dynamics of “territorialization” of the pandemic in the city of Rome was explored. The first of these dimensions, the spatial one, has been probed in terms of interaction between the UAs, an interaction which is assumed to be stronger the greater their territorial contiguity. The second aspect, the temporal one, is analyzed in terms of differentials in the growth rates of infections between two moments in time. Furthermore, some information relating to the demographic structure and the territorial partition of the metropolitan territory has been used.

Particular attention is devoted to the elderly population—the most vulnerable to the consequences of the infection—which seems to play a significant role to block, or at least contain, the growth of infections. While, on the contrary, the density of children–young people and that of infections per km^2^ seem to show their significant role in the spreading of COVID-19.

Nevertheless, the most interesting information on spatial spillovers and the speed of infection between territorial units is given by the autoregressive effects of TVI and the temporal effects of the IC. In fact, it would seem that the increase in cases of infections in the analyzed period is not of an intensive type (increase in infections in the UAs where they were already high), but extensive.

The indirect and total impacts of the spatial delay of the explanatory variables show the presence of a territorial framework between the UAs which can be interpreted as the core of the spatial relationships between the various areas of the city, highlighting both the local and global ones. It is thanks to this framework that it is possible to explore the effects of the interaction between the epidemic (bio) and the city (society).

## Data availability statement

Publicly available datasets were analyzed in this study. This data can be found here: https://www.deplazio.net.

## Author contributions

FRL and FGT contributed to conceptualization, investigation, and writing of original draft preparation. FGT contributed to methodology, formal analysis, and data curation. FRL contributed to supervision, visualization, and writing of review and editing. Both authors have read and agreed to the published version of the manuscript.

## Funding

This study was funded by Ministry of University and Research Grant PRIN 2020NCKXBR.

## Conflict of interest

The authors declare that the research was conducted in the absence of any commercial or financial relationships that could be construed as a potential conflict of interest.

## Publisher's note

All claims expressed in this article are solely those of the authors and do not necessarily represent those of their affiliated organizations, or those of the publisher, the editors and the reviewers. Any product that may be evaluated in this article, or claim that may be made by its manufacturer, is not guaranteed or endorsed by the publisher.

## Footnotes

^1^The theories of Agamben (2020) on the “health dictatorship” are certainly more complex than those reported by the mass media and, in particular, those disseminated on social networks. The strong and harsh reactions that they provoked, perhaps, are mainly due to the fact that Agamben's reflections provided support and reinforced the theories of the so-called “negationist front” in which the role of the far right neo-fascist origin is by no means marginal.

^2^The shift of attention from the centrality of the territory to the population is the basis for the theory of the pastoral power which will be discussed in the following pages.

^3^An interesting book (see the bibliography hereunder), by the group of #mapparoma (https://www.mapparoma.info/), is devoted to the study of inequalities in the city of Rome.

^4^Among the different criteria (distance between centers of gravity, k-closest, etc.) that of physical proximity is the one that maximizes the spatial autocorrelation of the rate of variation.

^5^As noted by Anselin (1988), the SEM is equivalent to a special form of SAR also known as the Durbin spatial model which is derived by transforming the dependent and explanatory variables: (Y - λWY) e (X - λWX); so, we have Y = λWY + Xβ - λWXβ + ξ. This model is derived from the more general one: Y = λWY + Xβ + WXγ. The common factor test verifies the null hypothesis Ho: γ = -λβ; the rejection of the null hypothesis implies the rejection of the SEM specification in favor of a broader model with spatially delayed explanations.

^6^In order to give a correct interpretation to the effects of these variables, it is necessary to make a brief clarification. Both subdivisions are calculated with two binary variables which can, as already remarked, assume values equal to 1 or 0 depending on whether the UA belongs, or not, to a territorial partition. Therefore, the effects of the spatial delay, which is given by the average assumed by the variable being analyzed in the UA neighboring the *i*-th UA, can be explained as follows:

$$\begin{array}{l} Wx_{i}=\;\frac{\sum_{j}w_{ij}x_{j}}{n} \end{array}$$

This figure is nothing more than the proportion of UA contiguous to the *i*-th UA, for which Wx_i_ can only assume values between 0 and 1. Therefore, the spatial delay is nothing more than an average of these values which is hence the proportion of UA neighboring the *i*-th UA belonging to the same territorial partition.

^7^The Moran index reports whether and to what extent neighboring territorial units are similar/different in relation to the intensity of a certain phenomenon and can be formulated in this way (Truglia, 2011):

$$\begin{array}{l} I=\frac{n\sum_{i}\sum_{j}\left(x_{i}-M\right)\left(x_{j}-M\right)w_{ij}}{\sum_{i}\sum_{j}w_{ij}\sum_{i}{\left(x_{i}-M\right)}^{2}} \end{array}$$

where - x_i_ and x_j_ are the intensities of the examined variable (in this case TVI, IV, and D) in the *i*-th zone and the *j*-zones close to it; - M is the arithmetic mean of the variable; - w_ij_ is the element of the contiguity matrix W; and - n the number of territorial units. In general, the index I varies between −1 and 1. In the first case, there is negative autocorrelation, contiguous areas have different values of the variable x_i_, while non-contiguous areas have similar values of x_i_. In the second case, on the contrary, there is a positive autocorrelation for which contiguous areas report similar values of the variable under study. In the case of the absence of autocorrelation, the index value is equal to −1/n-1.

^8^The analogy, between the human body and that of the sovereign, forms the iconoclastic imagine of power, starting with the image on the cover of Leviathan. However, the genesis of this identification is older and, certainly, it already played a fundamental role in the political theology of Saint Paul which was aimed at founding a deep-rooted order, but capable of lasting on earth. On the relationship between the believer's body and the body of Christ, see Taubes (1993), translation into Italian 1997. On the other hand, Canetti, describes in three paragraphs *The grasping and incorporating, The hand*, and *On the psychology of eating*, the relationship between these parts or functions of the body and the organs of power (Canetti, 1960, pp. 243-269).

^9^In this regard, think of the possibility of pooling vaccines against COVID-19 and the biopolitical implications of this choice.
